# Detection and surveillance of circulating tumor cells in osteosarcoma for predicting therapy response and prognosis

**DOI:** 10.20892/j.issn.2095-3941.2022.0279

**Published:** 2022-09-23

**Authors:** Haoran Mu, Dongqing Zuo, Jie Chen, Zhigang Liu, Zhuo Wang, Liu Yang, Qihui Shi, Yingqi Hua

**Affiliations:** 1 Shanghai Bone Tumor Institute and Department of Orthopedics, Shanghai General Hospital, Shanghai Jiao Tong University School of Medicine, Shanghai 200080, China; 2 Key Laboratory of Systems Biomedicine (Ministry of Education), Shanghai Center for Systems Biomedicine, Shanghai Jiao Tong University, Shanghai 200240, China; 3 Shanghai Key Laboratory of Medical Epigenetics and the International Co-laboratory of Medical Epigenetics and Metabolism (MOST), Institutes of Biomedical Sciences, Fudan University, Shanghai 200032, China; 4 Key Laboratory of Whole-Period Monitoring and Precise Intervention of Digestive Cancer (SMHC) and Institute of Fudan-Minhang Academic Health System, Minhang Hospital, Fudan University, Shanghai 201199, China; 5 Shanghai Engineering Research Center of Biomedical Analysis Reagents, Shanghai 201203, China

**Keywords:** Circulating tumor cells, osteosarcoma, hexokinase 2, single-cell sequencing, mesenchymal tumor

## Abstract

**Objective:**

Osteosarcoma (OS) is an aggressive, highly metastatic, relatively drug-resistant bone tumor with poor long-term survival rates. The presence and persistence of circulating tumor cells (CTCs) in the peripheral blood are believed to be associated with treatment inefficiency and distant metastases. A blood-based CTC test is thus greatly needed for monitoring disease progression and predicting clinical outcomes. However, traditional methods cannot detect CTCs from tumors of mesenchymal origin such as OS, and research on CTC detection in mesenchymal tumors has been hindered for years.

**Methods:**

In this study, we developed a CTC test based on hexokinase 2, a metabolic function-associated marker, for the detection and surveillance of OS CTCs, and subsequently explored its clinical value. Twelve patients with OS were enrolled as the training cohort for serial CTC tests. Dynamic CTC counting, in combination with therapy evaluation and post-treatment follow-up, was used to establish a model for predicting post-chemotherapy evaluation and disease-free survival, and the model was further validated with a cohort of 8 patients with OS.

**Results:**

Two dynamic CTC number patterns were identified, and the resulting predictive model exhibited 92% consistency with the clinical outcomes. This model suggested that a single CTC test has similar predictive power to serial CTC analysis. In the validation cohort, the single CTC test exhibited 100% and 87.5% consistency with therapy response and disease-free survival, respectively.

**Conclusions:**

Our non-invasive test for detection and surveillance of CTCs enables accurate prediction of therapy efficiency and prognosis, and may be clinically valuable for avoiding inefficient therapy and prolonging survival.

## Introduction

Osteosarcoma (OS), the most common primary bone tumor, tends to occur in children, adolescents and young adults, with an annual overall incidence rate of ~3 per million^1^. OS usually occurs in the metaphysis with abundant blood supply, thus leading to high rates of early metastasis and poor prognosis^2^. Surgery combined with chemotherapy is the standard treatment for OS, and the overall 5-year survival rate is 60%–70%^3^. However, patients who respond poorly to chemotherapy have high incidence of recurrence or metastasis, with 5-year survival rates of ~20%^3^. Thus, development of a rapid and accurate surveillance method is urgently needed. Liquid biopsy, an emerging technology, provides a new way to monitor disease and predict whether a patient will respond to chemotherapy or develop metastases; this ability would clearly improve treatment strategies and patient outcomes.

Hematogenous metastasis of OS cells results in recurrence or distant metastasis. Circulating tumor cells (CTCs) are tumor cells that slough off the primary tumor, and extravasate into and circulate in the blood before metastases develop^4,5^; therefore, they are an ideal marker for detecting potential metastases. CTCs are natural targets of antitumor treatments, and their persistence in the peripheral blood clearly indicates treatment inefficiency. Thus, developing a CTC-based surveillance test would greatly facilitate the prediction of OS therapy response and prognosis.

Although many methods are available for CTC detection, they are based mainly on epithelial markers (e.g., EpCAM and cytokeratin) and are inherently biased toward identifying CTCs with epithelial traits^6,7^. Current methods are rarely efficient in the detection of CTCs with aberrant activation of epithelial-mesenchymal transition (EMT) or those from tumors of mesenchymal origin^7,8^. Few studies have examined CTC detection in mesenchymal malignancies (e.g., sarcomas or melanoma)^8–11^. For OS, the marker of cell-surface vimentin and size selection membranes have been used to detect CTCs in a very limited number of patients; however, the detection rates are low^9,10^. Metastasis associated protein 1 (MTA1) has also been investigated in several tumors, including OS, and its overexpression has been established to be associated with high-risk characteristics of OS. However, MTA1 is not expressed in all CTCs, and the rates of MTA1 expression across CTC phenotypes significantly differ^11^. Thus, MTA1 is not a perfect marker for CTC detection. Other markers, such as type I collagen, lack specificity, and baseline expression of these markers is observed on non-tumor cells^12^. For many reasons, research on the detection of CTCs from mesenchymal malignancies has been hindered for years. Developing new methods and exploring their clinical applications are urgently needed.

In our previous studies, we developed a hexokinase 2 (HK2)-based test for detecting rare tumor cells in multiple body fluids (e.g., pleural effusion, urine, blood, and cerebrospinal fluid) and cancers^13–16^. HK2 is associated with metabolic reprogramming, a hallmark of cancer^17,18^. HK2, which catalyzes the first committed step in glucose metabolism, is induced in cancer cells. Its role in tumorigenesis has been attributed to its glucose kinase activity^19^. HK2 expression is higher in patients with metastatic tumors than primary tumors^13,20,21^, and is associated with advanced tumor grades^21^. High expression of HK2 in tumor tissues is critical for the proliferation, migration, and chemoresistance of tumor cells^20–22^. This functional marker enables the detection of CTCs with epithelial traits and EMT, particularly cytokeratin negative CTCs, on the basis of our recent studies^13–16^. Because OS is metabolically driven by aerobic glycolysis^23,24^, HK2 may be a suitable CTC marker for OS. In this study, we investigated the utility of an HK2-based test for detection and surveillance of CTCs in patients with OS, and explored its clinical value for predicting therapy response and prognosis.

## Materials and methods

### Patient information and sample collection

The protocol was approved by the institutional ethics committee at Shanghai General Hospital (2017KY017, 2019SQ268) and was conducted according to the principles of the Declaration of Helsinki. Informed consent from each participant was obtained. Peripheral blood and tissue samples were obtained from patients with OS who had provided written informed consent in Shanghai General Hospital from 2018 to 2020. These patients were followed up until recurrence/metastasis or 24 months after chemotherapy. The healthy volunteers whose blood was tested had no known illness or fever at the time of the blood draw and no history of malignant diseases.

### Fabrication of a microwell chip

The microwell chip was fabricated in poly(dimethylsiloxane) (PDMS) through standard microfabrication soft-lithographic techniques. The PDMS pre-polymer (Sylgard 184, Dow Corning, USA) was mixed in a ratio of 10:1 and cast on a lithographically patterned SU-8 2050 replicate. After curing at 80 °C for 2 h, the PDMS component was separated from the replicate. The PDMS microwell chip contained 112,000 microwells with 30 μm diameter and 20 μm depth.

### Blood sample processing and the HK2-based CTC test

Fresh peripheral blood samples (5 mL) were drawn and preserved in TransFix/EDTA Vacuum Blood Collection Tubes. The samples were delivered to the laboratory within 4 hours after the blood draw. Blood samples were mixed with 25 μL CTC enrichment antibody cocktail (RosetteSep™ CTC Enrichment Cocktail Containing Anti-CD36) at room temperature for 20 min, and 15 mL HBSS with 2% FBS (Gibco) was then added to the sample and mixed well. The mixture was carefully added along the wall of a SepMate tube (SepMate™-50) after addition of 15 mL of density gradient liquid (Lymphoprep™) into the tube. After centrifugation at 1200 g for 20 min, the supernatant (~10 mL) was discarded, and the remaining liquid (~10 mL) above the barrier of the SepMate tube was rapidly poured into a new centrifuge tube. After centrifugation at 600 g for 8 min, the supernatant was removed, and 1 mL of red blood cell lysis buffer (BD Biosciences) was added and allowed to stand for 5 min to lyse red blood cells. After centrifugation at 250 g for 5 min, the nucleated cell pellet was re-suspended in HBSS. The cell suspension was then applied onto the 3% BSA-treated microwell chip. Cells were allowed to stand for 10 min in the 112,000 microwells. After cell fixation (2% PFA, 10 min) and permeabilization (0.5% Triton X-100, 15 min), blocking solution consisting of 3% BSA and 10% normal goat serum was applied to the chip for 1 h, and the chip was then incubated with APC-conjugated anti-CD45 antibody (mouse) and anti-HK2 antibody (rabbit) in PBS overnight at 4 °C. After extensive washing with PBS, cells on the chip were treated with Alexa Fluor 488-conjugated goat-anti-rabbit secondary antibody in PBS for 1 h and DAPI for 10 min, then washed with PBS. An ImageXpress Micro XLS field High Content Screening System (Molecular Devices) was used to scan the chip and take images of cells in bright field and three fluorescent colors (CD45: CY5; HK2: FITC; nuclei: DAPI). A deep learning-based AI algorithm identified HK2^high^/CD45^－^/DAPI^+^ cells as putative CTCs. The HK2^high^ cutoff was generated from the HK2 fluorescence intensity of CD45^+^ leukocytes in the samples.

### Single-cell low-depth whole genome sequencing

For characterizing single-cell copy number profiles, we performed genome amplification on retrieved single cells with a MALBAC^®^ Single Cell WGA Kit (Yikon Genomics, China), and next-generation sequencing (NGS) libraries were then constructed with an NEBNext^®^ Ultra™ DNA Library Prep Kit for Illumina (New England Biolabs). Libraries were sequenced on the Illumina HiSeq X Ten platform (Genewiz, China). FASTQ files were aligned to the major chromosomes of the human genome (hg19) with BWA (version 0.7.10-r806). SAMtools (version 1.3.1) was used to convert SAM files to BAM files and to remove PCR duplicates. Aligned reads were counted in fixed bins averaging 500 kb. GC content was used to normalize sequence depths. The diploid regions were determined with HMMcopy. Segmentation was performed with the circular binary segmentation method (alpha=0.0001 and undo. prune = 0.05) in the R Bioconductor ‘DNAcopy’ package.

### Cell lines, patient-derived xenograft (PDX) models, and reagents

The OS cell lines U-2 OS, SJSA-1, 143B, HOS, and MG63 were purchased from the American Type Culture Collection (ATCC, USA) and were maintained in modified McCoy’s 5a medium, RPMI-1640 medium, and Eagle’s minimum essential medium, respectively, supplemented with 10% fetal bovine serum (Gibco, USA) and 1% penicillin-streptomycin solution (Invitrogen, USA) at 37 °C in a humidified incubator containing 5% CO_2_. The PDX models were established with surgical specimens from patients with OS. Each specimen was cut into 2 mm × 2 mm sections in tissue culture medium under aseptic conditions and then subcutaneously implanted into the BALB/C-nu mice (Shanghai SLAC Laboratory Animal, Shanghai, China). APC-conjugated CD45 (clone HI30), Alexa Fluor 488-conjugated goat-anti-rabbit secondary antibody (#A11008), and MitoTracker™ Green FM (#M7514) were purchased from Thermo Fisher Scientific. Anti-HK2 primary antibody was purchased from Abcam (#ab209847). DAPI was purchased from Beyotime Biotechnology. Hanks’ balanced salt solution (HBSS, no calcium, no magnesium, no phenol red) was purchased from Gibco. RosetteSep™ CTC Enrichment Cocktail Containing Anti-CD36, SepMate™-50 SepMate tubes and Lymphoprep™ density gradient solution were purchased from STEMCELL Technologies.

### Statistical analysis

Statistical analyses were performed in GraphPad Prism 8 (GraphPad Software, Inc.). Data are reported as mean ± standard deviation. The normality of the data was tested with the Kolmogorov-Smirnov test. Two-tailed Mann-Whitney and Kruskal-Wallis tests were performed as nonparametric tests between 2 or more groups that were not normally distributed, respectively. The Kaplan–Meier method was used to estimate the survival rate, along with a log-rank statistical test (two-sided) comparing the survival distribution.

## Results

### HK2-based CTC detection and assay validation

**Figure 1:**
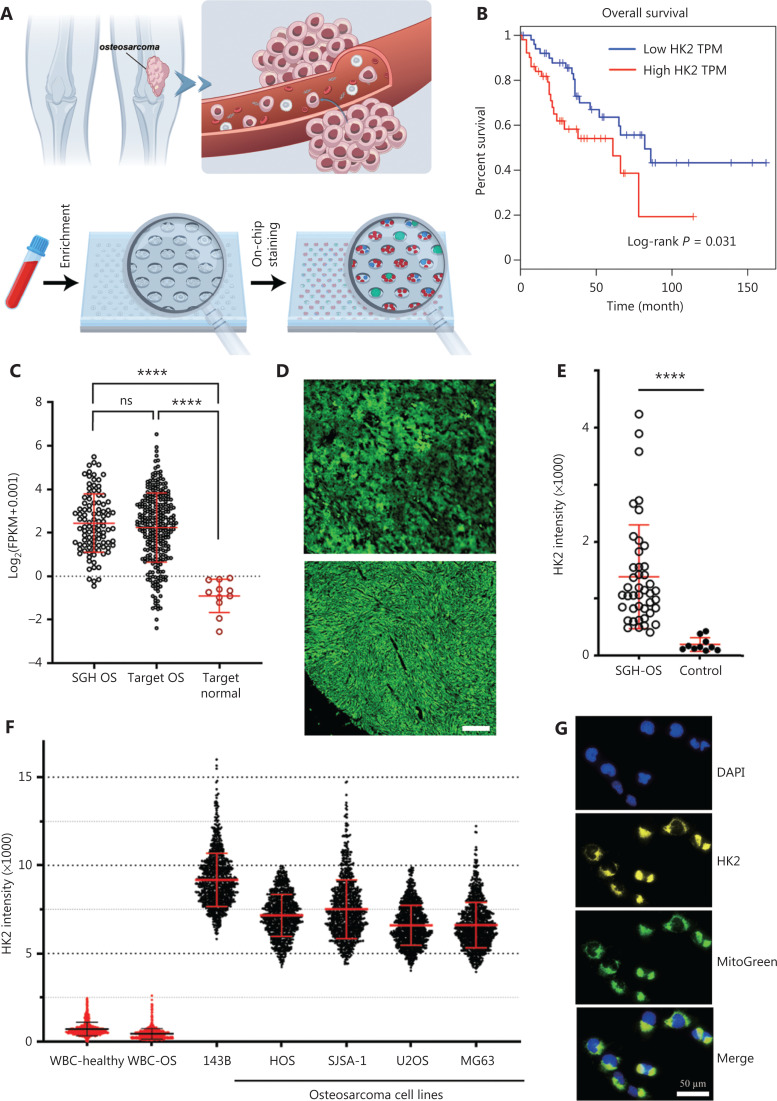
Overall strategy of HK2-based CTC detection and validation. (A) Schematic illustration of CTCs that are disseminated into the blood and initiate metastasis, and the workflow of CTC detection. (B) Kaplan-Meier survival curves showing significantly lower overall survival for high HK2 expression in OS. TPM: transcripts per million. (C) HK2 expression in patients with OS and normal controls from the SGH and TARGET cohorts. (D) Fluorescence images of HK2 levels in formalin-fixed and paraffin-embedded (FFPE) OS tumor tissues. Scale bar: 100 μm. (E) HK2 intensity of OS tumor tissues in the SGH cohort and normal controls. (F) HK2 intensity of OS cell lines and white blood cells (WBCs) from a healthy donor and a patient with OS. (G) 143B cells fluorescently stained with anti-HK2 antibody, a mitochondrial marker (MitoGreen), and DAPI.

OS usually occurs in the metaphysis with abundant blood supply and thus has high tendency to shed tumor cells into circulation and initiate metastasis. The workflow of CTC detection is shown in **Figure 1A**. Briefly, negative enrichment was first performed with a RosetteSep™ CTC Enrichment Mixture and density gradient centrifugation for depleting red blood cells and most leukocytes. A PDMS microwell chip was then used to accommodate nucleated cells for immunostaining of HK2 (metabolic marker), CD45 (leukocyte marker), and DAPI nuclear staining; this was followed by identification of putative CTCs (HK2^high^/CD45^−^/DAPI^+^ cells) with a deep learning-based algorithm (**Supplementary Figure S1**).

HK2, a metabolic function-associated marker, was used for detecting CTCs of OS on the basis of the following results. First, in The Cancer Genome Atlas (TCGA) cohort, patients with OS with high HK2 had lower overall survival than patients with OS with low HK2 expression (**Figure 1B**, log-rank *P*=0.031), thus indicating that HK2 levels were associated with prognosis of OS. Second, in the Shanghai General Hospital (SGH) and Therapeutically Applicable Research to Generate Effective Treatments (TARGET) cohorts, the high HK2 expression of patients with OS was found to be significantly higher than that in normal controls (**Figure 1C**). Likewise, the HK2 expression at the protein level in OS tissues from the SGH cohort was significantly higher than that in normal controls (**Figure 1D, 1E and Supplementary Figure S2**). Third, high HK2 expression was also found in OS cell lines but was rarely observed in leukocytes from the peripheral blood (**Figure 1F and Supplementary Figure S3**). Meanwhile, HK2 staining colocalized with mitochondrial staining (**Figure 1G**), in agreement with literature reports^11,12,15,16^.

**Figure 2:**
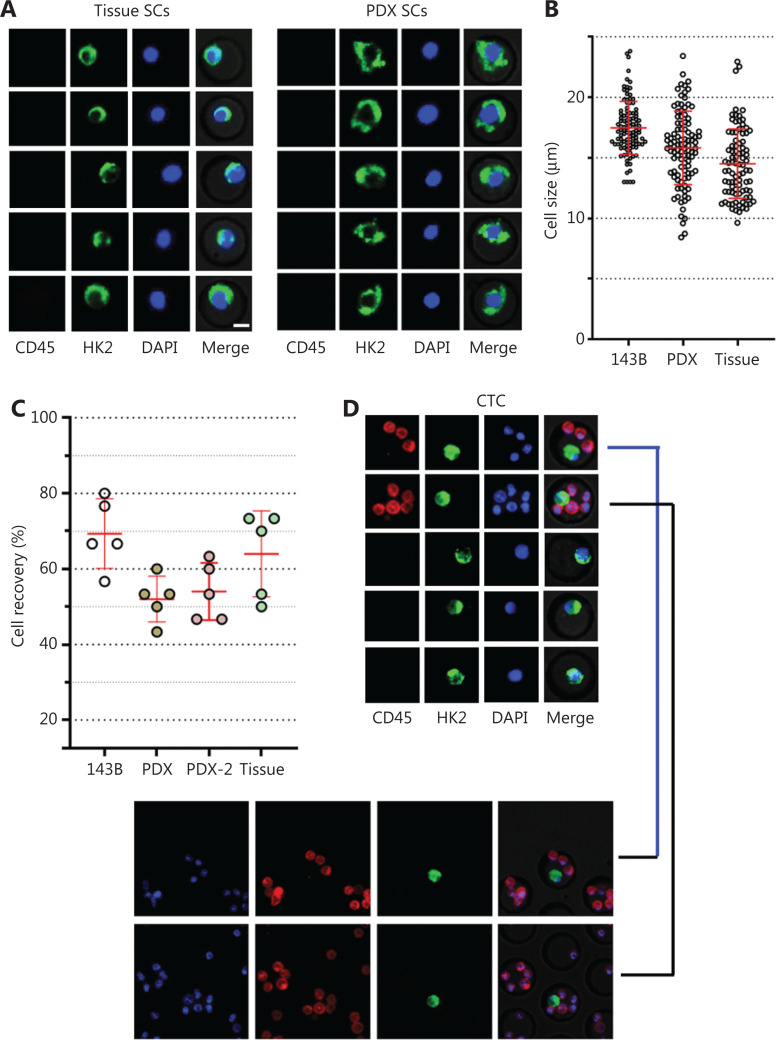

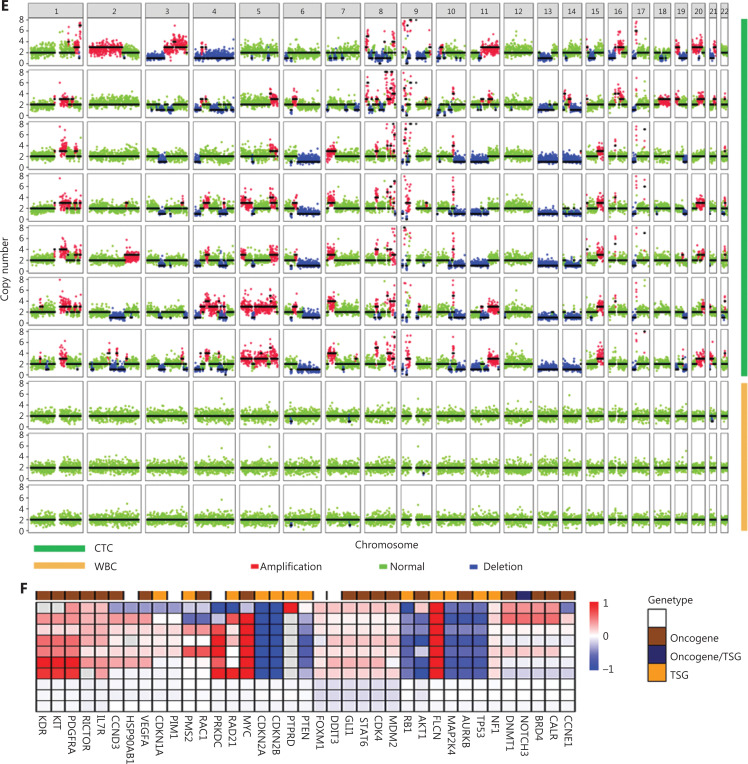

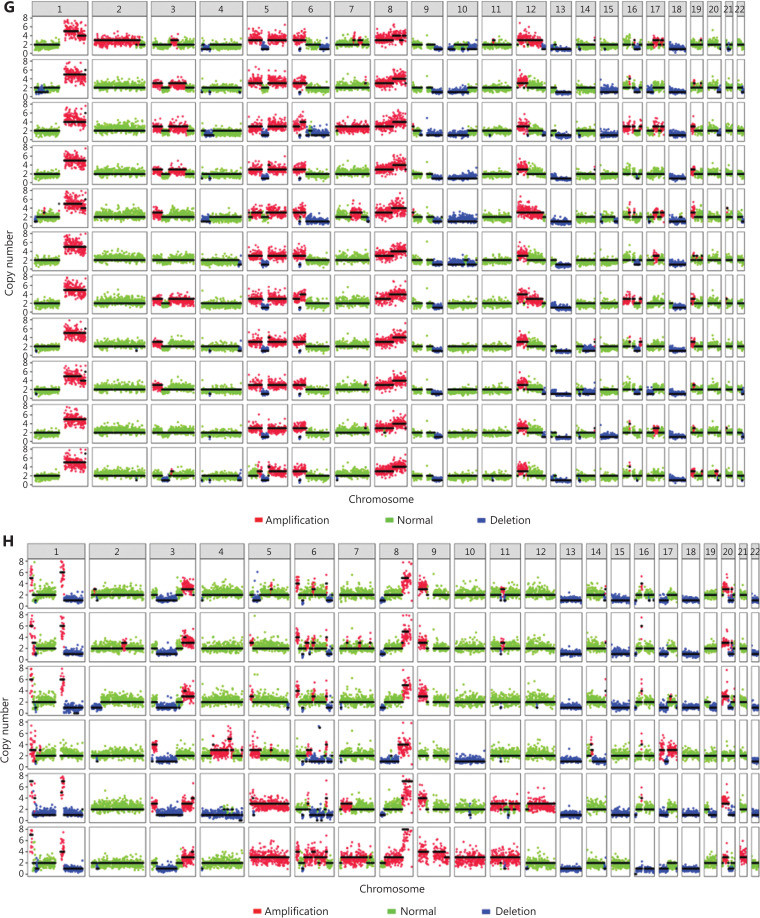
CTC recovery of the assay and the malignancy of CTCs detected by the HK2 test. (A) Fluorescence images of single cells digested from tumor tissues of a patient with OS and a PDX model. Scale bar: 10 μm; SC: single cell. (B) Sizes of tumor cells from the 143B cell line, and tumor tissues of a PDX model and a patient with OS. (C) CTC recovery of the spike-in experiments based on 143B cells, PDX SCs, PDX-2 SCs, and tissue SCs. (D) Fluorescence images of tissue SCs spiked into blood and detected by the HK2-based CTC test. Scale bar (E) Single-cell CNA profiles of 7 HK2^high^/CD45^－^/DAPI^+^ CTCs and 3 WBCs. (F) Heatmap of adjusted copy number gains (red) or losses (blue) of oncogenes and tumor suppressor genes (TSGs) across the genome within the cells shown in **Figure 2E**. Adjusted gene copy number = log_2_ (copy number) − 1. (G, H) Single-cell CNA profiles of PDX SCs and PDX-2 SCs spiked into the blood and detected by the HK2-based CTC test.

To demonstrate a proof of concept, we spiked 30 OS cells into 5 mL of blood from healthy donors to mimic blood samples from patients with OS, then performed HK2-based testing for CTC detection and recovery assessment. Four types of OS cells were used in the spike-in experiments: 143B (OS cell line), tissue SC (digested from tumor tissue of a patient with OS), PDX SC, and PDX-2 SC (digested from tumor tissues of PDXs; **Figure 2A and Supplementary Figure S4**). These OS cells were mostly larger than 10 μm (**Figure 2B**). After these OS cells were spiked into the blood, their recovery ranged from 53% to 80%, indicating moderate CTC loss in the HK2-based CTC assay (**Figure 2C**). **Figure 2D** shows representative images of CTCs detected from the tissue SC-spiked blood sample. To validate the malignancy of CTCs detected by the HK2 test, we randomly selected and sequenced CTCs. **Figure 2E** presents the single-cell copy number alteration (CNA) profiles of CTCs with leukocytes as the control. All sequenced CTCs exhibited reproducible copy number gains and losses across the genome and were distinct from CD45^+^ leukocytes with an absence of CNAs. The recurrent genomic aberrations in multiple cells were characteristic of malignant cells, thus indicating the high accuracy of the HK2 test in detecting CTCs of OS. CNAs of 37 genes frequently amplified or lost in OS are plotted in **Figure 2F**. Notable findings included the loss of the tumor suppressors *TP53*, *CDKN2A* (p16), and *CDKN2B* (p15), which found in all CTCs, as well as the amplification of the oncogene *MYC* in 6 of 7 CTCs. Likewise, CTCs detected from PDX SC- and PDX-2 SC-spiked blood samples were randomly sequenced (single-cell CNA profiles in **Figure 2G, 2H and Supplementary Figure S6**). Reproducible gains and losses in CNA patterns demonstrated that all HK2-derived CTCs were truly tumor cells.

### Strategy for establishing CTC-based predictive models

**Figure 3:**
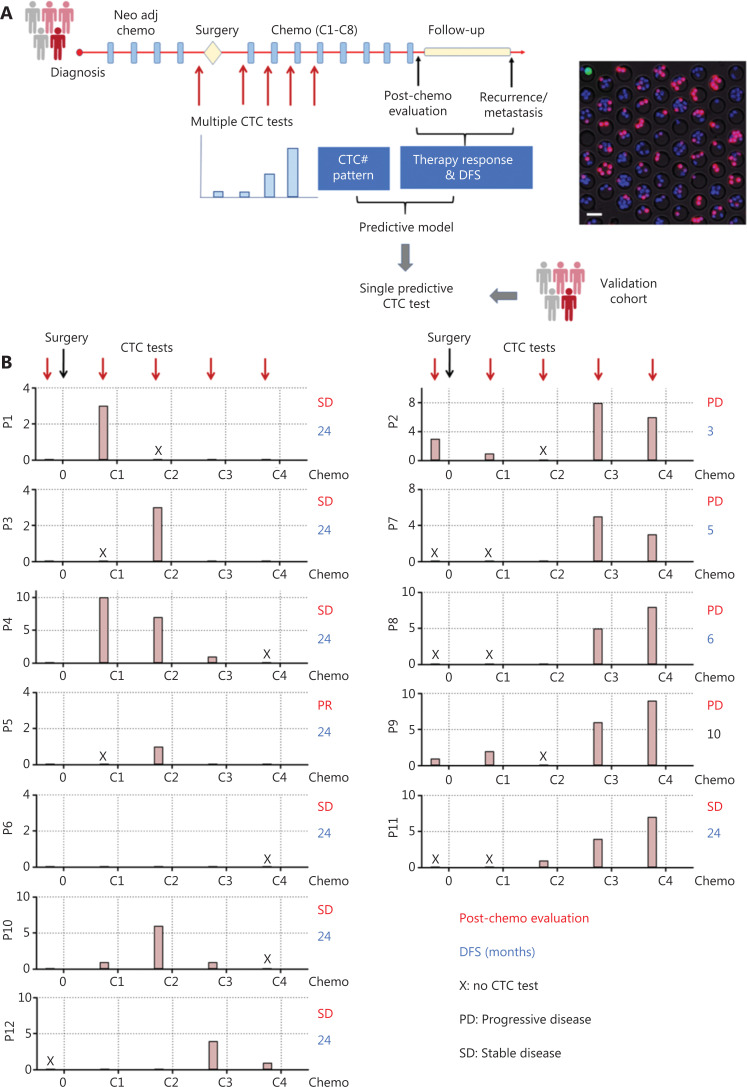
Strategy of establishing and verifying CTC-based predictive models. (A) Left, schematic illustration of serial CTC measurements for establishing a predictive model for patients with OS; right, fluorescence images of CTCs detected by the HK2-based CTC test. Scale bar (B) Dynamic CTC number profiles based on the HK2 test and clinical outcomes (post-chemotherapy evaluation and DFS) of 12 patients with OS during the treatment. The time points for CTC measurement were before surgery and C1–C4 (1^st^–4^th^ rounds of chemotherapy).

We hypothesized that dynamic changes in CTC numbers might predict chemotherapy response and the prognosis of patients with OS. To test this hypothesis, we enrolled 12 patients with OS (stage IIA–IVA; **Supplementary Table S1**) and collected 45 blood samples for performing multiple CTC tests (**Figure 3A**). As a standard treatment, 8 rounds of chemotherapy (C1–C8) were conducted on patients with OS after surgery. Serial CTC tests before the surgery, C1–C4, were then associated with chemotherapy response and disease-free survival (DFS), as assessed through consecutive radiological assessments, to generate a predictive model. We additionally identified the appropriate time to perform a single CTC test to predict clinical outcomes with comparable accuracy to serial CTC testing. A single CTC test is clearly more cost efficient and clinically applicable. To validate the model based on a single CTC test, we enrolled 8 patients with OS as a validation cohort, in which CTC measurements were performed, and therapy evaluation and DFS were assessed (**Figure 3A**).

### Serial CTC tests predict therapy response and DFS

We first measured 20 peripheral blood samples from healthy donors for the HK2-based CTC test. No samples showed positive CTC counts. The healthy cohort provided a baseline for OS patient measurement. Twelve patients with OS (P1–P12; **Supplementary Table S1**) were then enrolled as a training cohort, and a total of 45 blood samples were collected for serial CTC tests (before surgery, C1–C4), therapy evaluation, and post-treatment follow-up until recurrence/metastasis or 24 months after surgery. **Figure 3B** presents the CTC test results and clinical outcomes (therapy response and DFS) for each patient. Two dynamic CTC number patterns were identified to establish a predictive model that may inform clinical practice.

**Figure 4:**
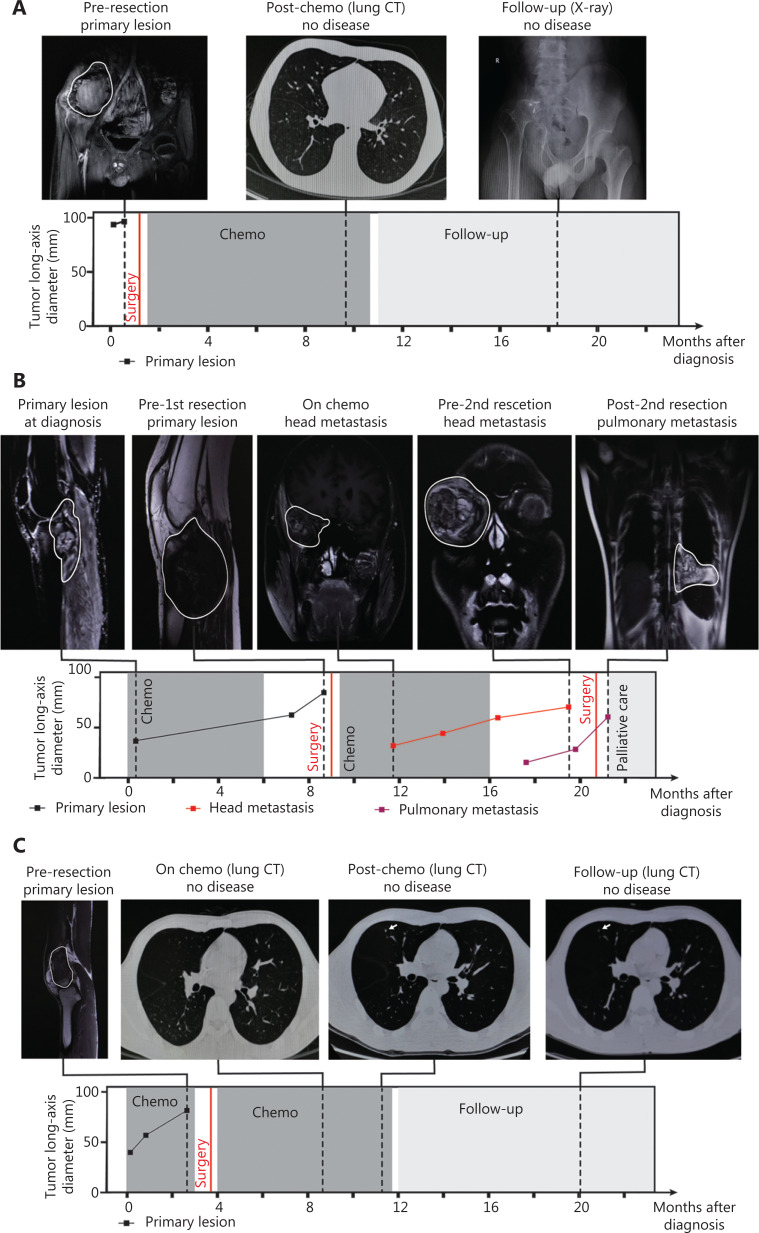
Relationship between CTC detection and patient clinical information. (A-C) Consecutive radiological assessments with response to treatment and follow-up in the management of P1, P4, and P2.

The first pattern was characterized by CTC numbers decreasing to 0 or 1 before C3 or C4, thus indicating a treatment response and favorable prognosis. This category included 7 patients (P1, P3, P4, P5, P6, P10 and P12) with a post-chemotherapy evaluation of partial response (PR) or stable disease (SD), as well as a DFS longer than 24 months. For example, P1 (stage IIIB) had positive CTC counting before chemotherapy, but the CTC number rapidly decreased to 0 before C3 (**Figure 3B**). No disease was found in the treatment and follow-up until 24 months (**Figure 4A**). Likewise, P4 (stage IIA) had a high CTC number before initiation of chemotherapy, but as the treatment progressed, the CTC number decreased 90%, to only 1, before C3, thus demonstrating efficient treatment and favorable prognosis (**Figure 4B**).

In the second pattern, CTC number increased to 5 or more before C3 or C4. The rapid increase and persistently high number of CTCs indicated high risk of hematogenous metastasis and poor prognosis. The cutoff number (5 CTCs per 5.0 ml peripheral blood) effectively distinguished unfavorable from favorable patient prognosis (**Supplementary Figure S7**). This category included P2, P7, P8 P9, and P11; P2, P7, P8, and P9 showed a post-chemotherapy evaluation of progressive disease (PD) and a DFS less than 1 year. For example, P2 (stage IVA) had positive CTC before surgery and chemotherapy, and the CTC number rapidly increased to 8 before C3 (**Figure 3B**). Head metastasis was observed at C4, thus resulting in a 3-month DFS and PD condition after chemotherapy (**Figure 4C**). In contrast to other patients in this category, P11 (stage IVA) was misclassified because of the SD condition in post-treatment assessment and a 24-month DFS. P11 had lung metastasis at the time of diagnosis, and a 2nd surgery was performed to resect lung metastasis after chemotherapy. The positive CTC counts during chemotherapy were attributable to the existing lung metastasis.

These 2 CTC number patterns established a serial CTC test-derived model enabling prediction of therapy response and prognosis. This serial CTC analysis-derived model showed 92% (11/12) consistency with the clinical outcomes. Of note, tumor stage is not an accurate marker for predicting therapy response, because half the stage III and IV patients in this cohort exhibited SD or PR condition after chemotherapy.

### Single HK2-based CTC test predicts therapy response and DFS

**Figure 5:**
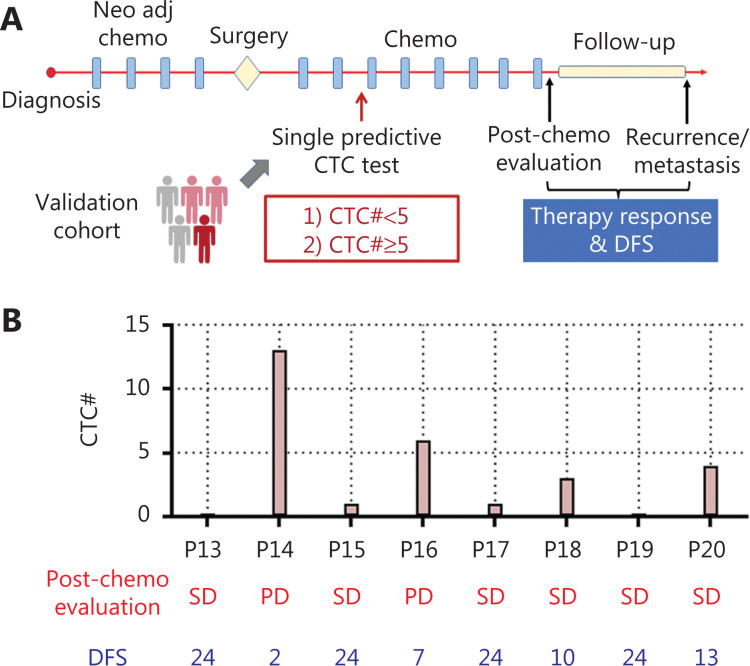
Single test-derived model for accurate prediction of therapy response and prognosis. (A) Schematic illustration of a single CTC test for predicting therapy response and prognosis in a validation cohort of patients with OS. (B) CTC test results and clinical outcomes of patients with OS in the validation cohort.

Compared with multiple CTC tests, a single CTC test is more desirable in clinical settings because it is cost-efficient and more acceptable to patients. The serial CTC analysis-derived model indicated that a single CTC test performed before C3 exhibited similar predictive power to multiple tests. Specifically, a CTC number ≥ 5 was associated with a post-treatment assessment of PD and short DFS (≤ 12 months), whereas a CTC number ≤ 1 indicated post-treatment assessment of SD or PR, and a long DFS (> 12 months). This single test-derived model led to 100% (12/12) consistency with the clinical outcomes in the training cohort. To further validate this model (**Figure 5A**), we enrolled 8 patients with OS as a validation cohort. The CTC test results and clinical outcomes (therapy response and DFS) are shown in **Figure 5B**. Among these 8 patients, P14 and P16 showed CTC counts greater than 5, which were associated with progressive disease after treatment and a short DFS (≤ 12 months). In contrast, P13, P15, P17, P19, and P20 had CTC numbers lower than 5, corresponding to a post-treatment assessment of SD and long DFS (> 12 months). P18 had a CTC number lower than 5 and SD in post-treatment assessment, but a short DFS (≤ 12 months). This single test-derived model exhibited 100% (8/8) and 87.5% (7/8) consistency with post-treatment assessment and DFS, respectively, thus demonstrating accurate prediction of therapy response and prognosis (**Supplementary Figure S7**).

## Discussion

Although its incidence is extremely low (approximately 3 per million), OS with high malignancy is more common in children, adolescents, and young adults than in older people. Despite intensive multi-agent chemotherapy, OS remains an aggressive, highly metastatic, and relatively drug-resistant tumor with poor long-term survival rates. A blood-based surveillance test for predicting therapy efficiency and disease progression would aid in the selection of appropriate treatment strategies and improve clinical outcomes. CTCs are ideal markers for developing a non-invasive test because their presence and persistence in the peripheral blood indicates inefficient therapy and potential distant metastases. Dynamic surveillance of CTCs in treatment can indicate whether a patient will respond to chemotherapy or develop metastases. However, current methods for CTC detection have failed to detect CTCs from tumors of mesenchymal origin, such as OS, because most methods use epithelial markers for CTC detection. Thus, research on CTC detection in mesenchymal tumors has remained hampered for years.

In this study, we developed a CTC test for detection and surveillance of CTCs in patients with OS, by using HK2 as a metabolic function-associated marker. HK2 enables detection of a broad spectrum of CTCs with elevated glycolysis, including epithelial CTCs, CTCs with aberrant activation of EMT, and CTCs from tumors of mesenchymal origin. We spiked OS cells from cell lines, and tumor tissues from PDXs and patients with OS, into the blood and evaluated the CTC capture efficiency of the microwell-based HK2 test. The HK2-dervied CTCs were validated with single-cell sequencing, and all showed recurrent CNVs across the genome—a characteristic of malignant cells. The spike-in experiments demonstrated the efficiency of HK2 for detecting CTCs in OS.

We investigated whether dynamic CTC tests might provide a novel indicator to predict therapy resistance and potential distant metastases. To this end, we enrolled 12 patients with OS in a training cohort and performed serial CTC tests before surgery and C1–C4 (the first half of chemotherapy). A predictive model was then established by comparison of the results of serial CTC tests with post-chemotherapy evaluation and DFS, on the basis of consecutive radiological assessments. Serial CTC analysis clearly predicted therapy efficiency and clinical progression, thus leading to 92% consistency with clinical outcomes including post-treatment assessment and DFS. Meanwhile, this serial CTC analysis-derived model indicated that a single CTC test was capable of predicting therapy response and prognosis with 100% consistency in clinical outcomes in the training cohort. The single CTC test was further validated with a validation OS cohort, and the prediction showed high consistency with clinical outcomes. The cutoff number for our single CTC test was 5, similar to the value of 7 in a previous study^11^. The slight difference might have been because our model confined the sampling time to just before C3. As shown in the serial CTC tests, different sampling times led to different results and consequently different cutoff numbers for CTCs. This finding indicates the importance of the sampling time, and highlights the value of our study. The limitation of this study is the small cohort size, because OS has an extremely low incidence (about 3 per million). For most of patients, the study lasted more than 30 months. The clinical utility of the HK2-based CTC test in OS still warrants a large-scale, multicenter clinical trial.

## Conclusions

We report a blood-based CTC test of OS, a mesenchymal bone tumor, that uses HK2 as a marker for detecting CTCs according to their metabolic abnormalities. Detection and surveillance of CTCs as a non-invasive test allows for accurate prediction of therapy efficiency and prognosis, thus enabling avoidance of inefficient therapy and improved survival.
