# Supplementary material for: Detection and surveillance of circulating tumor cells in osteosarcoma for predicting therapy response and prognosis
Source: Cancer Biol Med. 2022 Sep 23;19(9):1397–409. doi: 10.20892/j.issn.2095-3941.2022.0279 (PMC9500224; doi:10.20892/j.issn.2095-3941.2022.0279)
Supplement: Supplementary file 1 [file cbm-19-1397-s001.pdf]

Supplementary materials

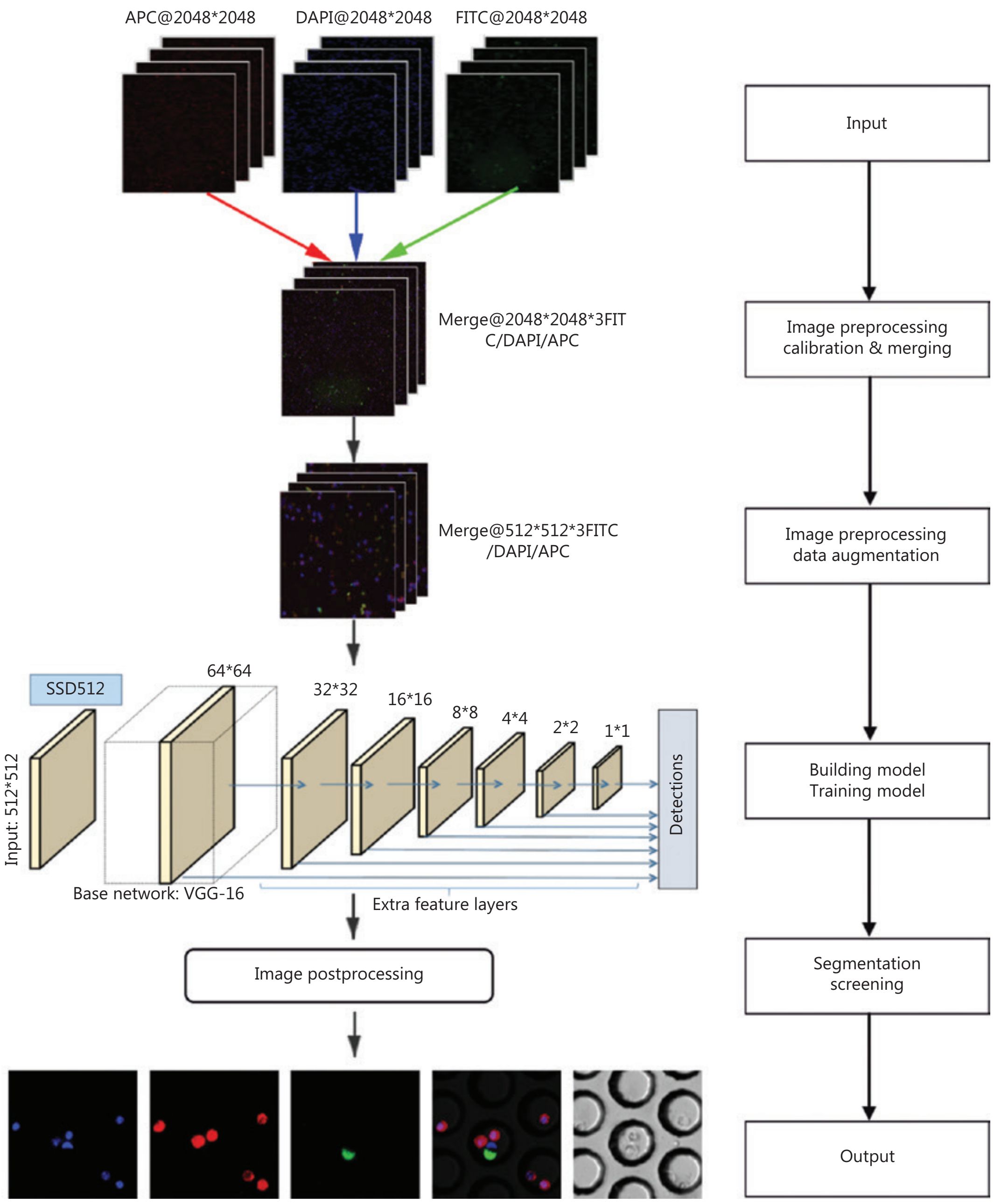

Figure S1 A deep learning-based algorithm to analyze images and identify putative CTCs ($HK2^+$/$CD45^-$/$DAPI^+$).

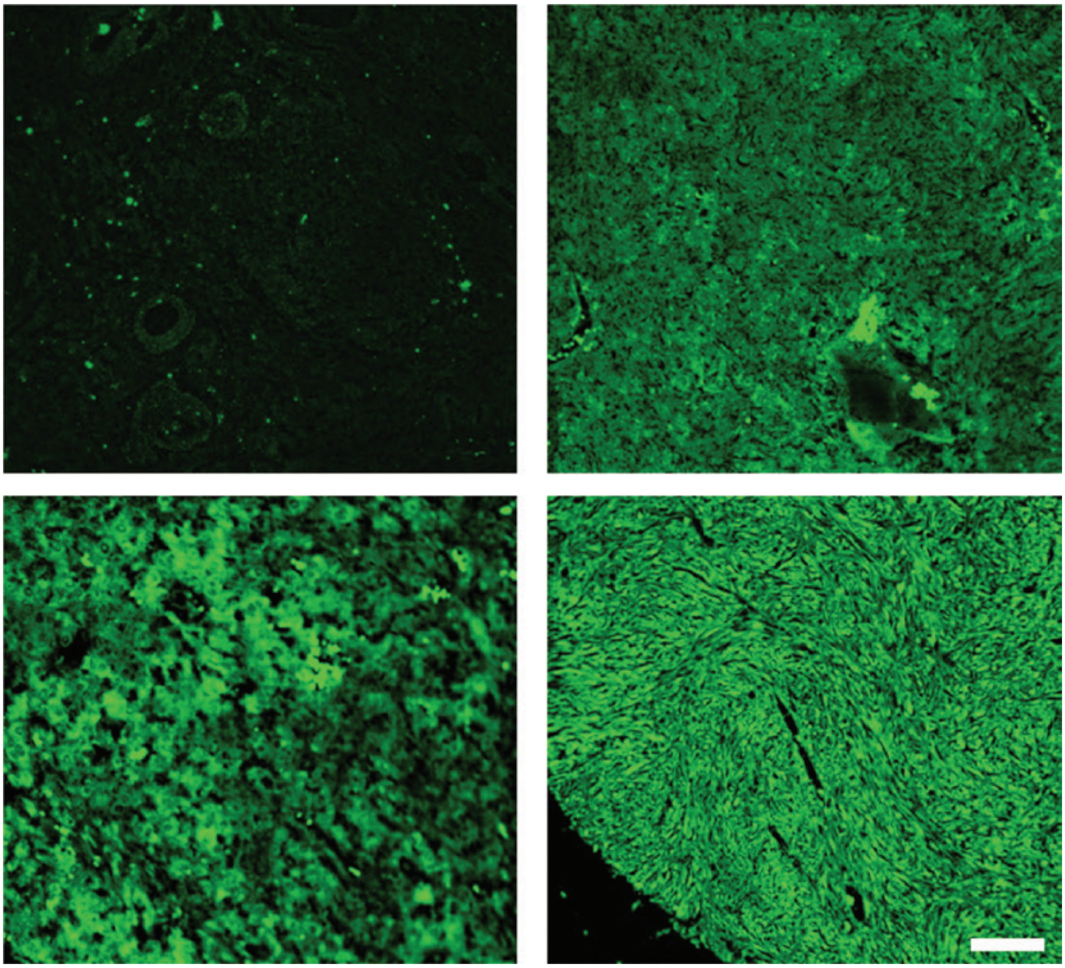

Figure S2 Fluorescence images of HK2 levels in formalin-fixed and paraffin-embedded (FFPE) OS tumor tissues from Shanghai General Hospital. Scale bar: 100 µm.

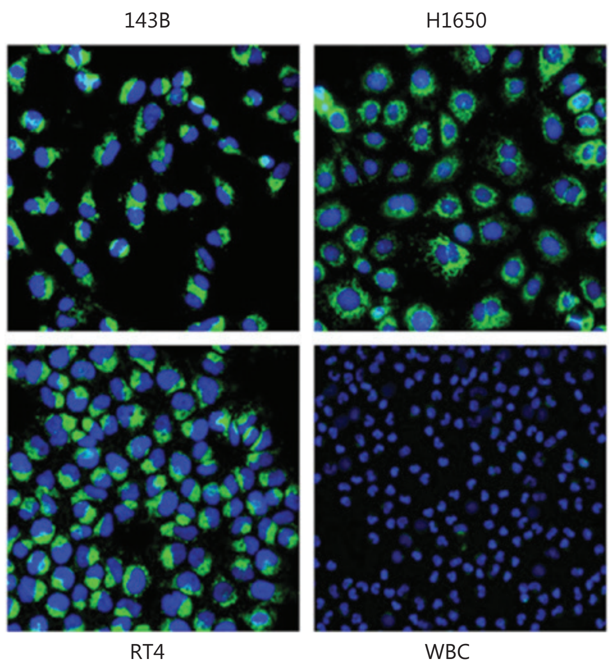

Figure S3 Top, representative fluorescence images of HK2 stained 143B, H1650 (lung cancer), and RT4 (bladder cancer) cells, and white blood cells (WBCs) from a healthy donor.

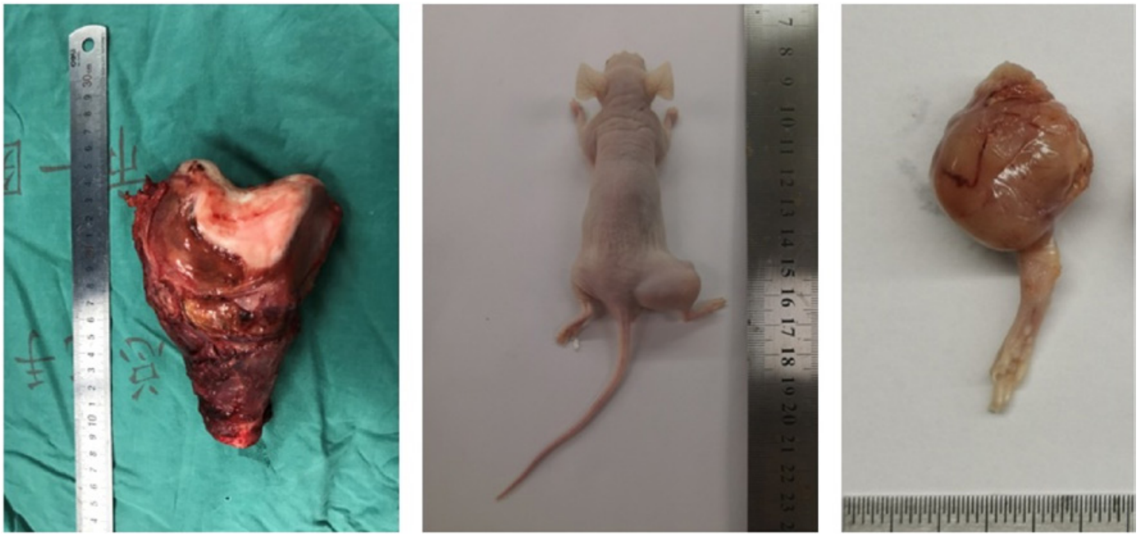

Figure S4 Left, resected osteosarcoma tumor; middle, osteosarcoma PDX model; right, resected tumor from the osteosarcoma PDX model.

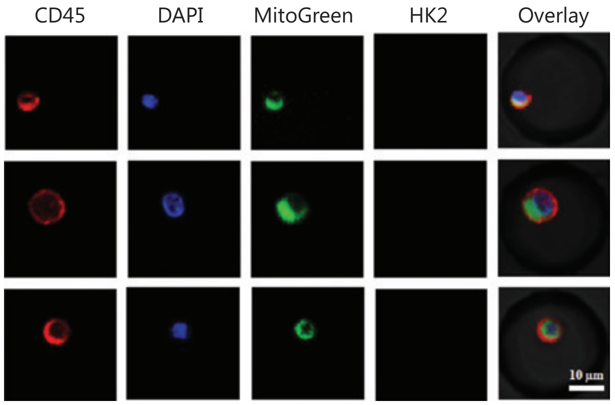

Figure S5 Representative fluorescence images of WBCs stained with CD45, HK2, and MitoGreen.

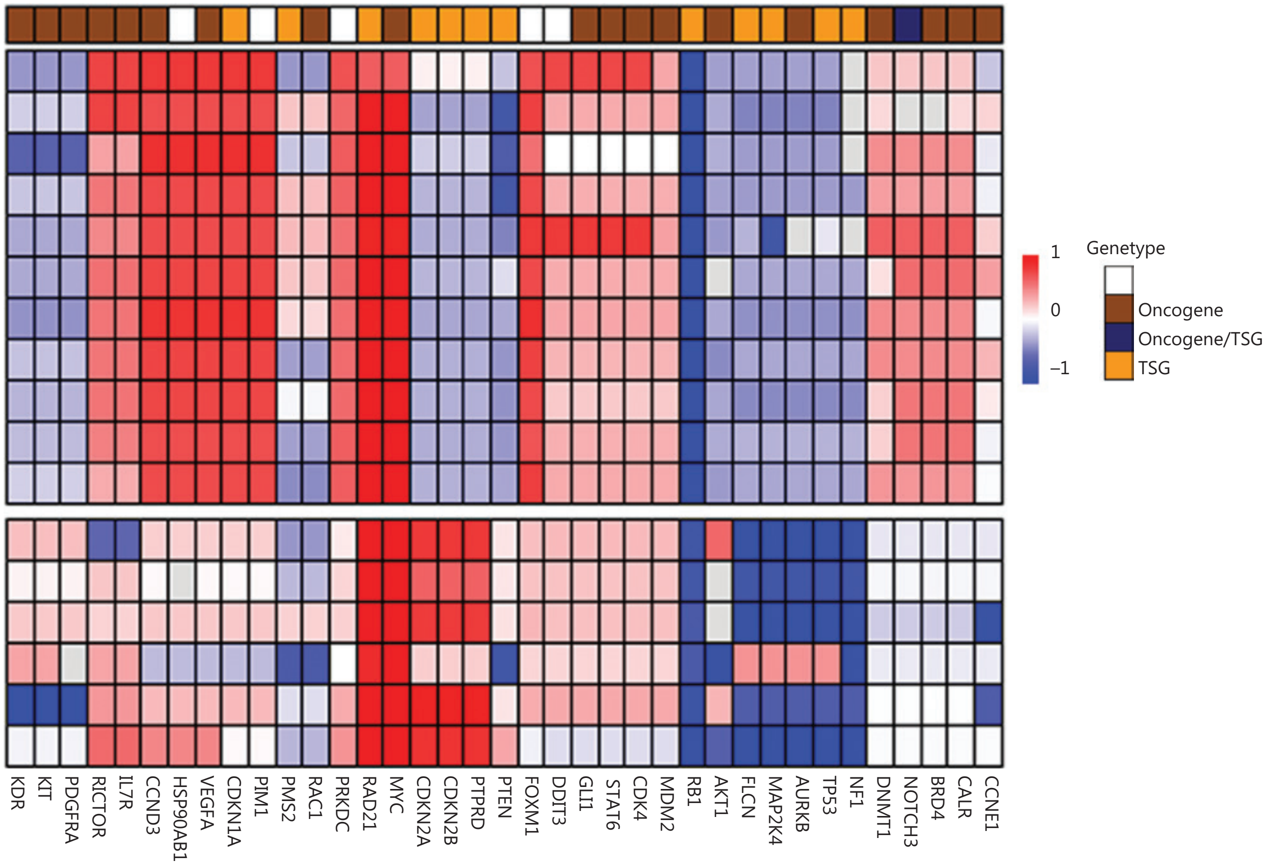

Figure S6 Heatmap of adjusted copy number gains or losses of oncogenes and tumor suppressor genes (TSGs) across the genome within the cells shown in **Figure 2G and 2H**. Adjusted gene copy number = $\log_2$(copy number) − 1.

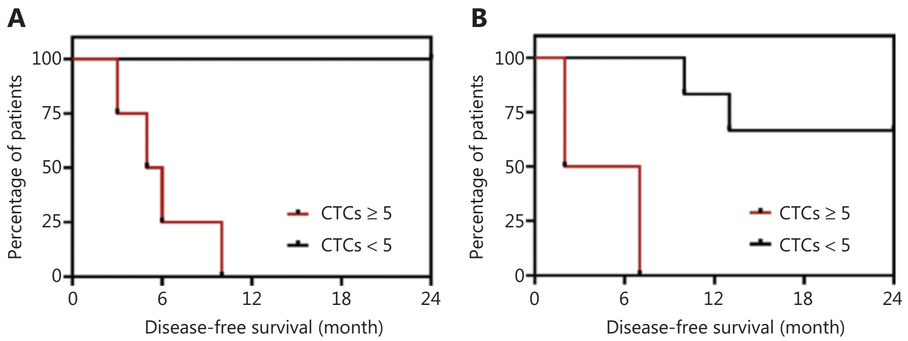

Figure S7 Kaplan-Meier plots of disease-free survival in patients with OS, with a cutoff of 5 CTCs per 5.0 mL peripheral blood detected before C3. (A) Serial CTC tests (*P* value: 0.0002). (B) Single CTC test (*P* value: 0.0039).

Table S1 Clinicopathological characteristics of patients with osteosarcoma in this study. SD: stable disease; PD: progressive disease; PR: partially response. *Therapy response was evaluated after chemotherapy. **DFS is defined as the period from surgery to metastasis/recurrence or follow-up to 24 months.

No.	Age	Gender	Stage	Therapy response* (months)	DFS**
P1	14	F	IIIB	SD	24
P2	23	M	IVA	PD	3
P3	33	F	IVA	SD	24
P4	19	M	IIA	SD	24
P5	14	F	IIA	PR	24
P6	21	M	IIA	SD	24
P7	15	F	IIB	PD	5
P8	36	F	IIIB	PD	6
P9	48	M	IIIA	PD	10
P10	21	M	IIA	SD	24
P11	25	F	IVA	SD	24
P12	18	M	IIB	SD	24
P13	15	M	IIA	SD	24
P14	14	F	IIIA	PD	2
P15	58	M	IIIB	SD	24
P16	19	M	IIB	PD	7
P17	38	F	IIA	SD	24
P18	29	M	IIB	SD	10
P19	14	M	IIB	PR	24
P20	41	M	IIB	SD	13
